# Spatiotemporal patterns of early afterdepolarizations underlying abnormal T-wave morphologies in a tissue model of the Purkinje-ventricular system

**DOI:** 10.1371/journal.pone.0280267

**Published:** 2023-01-09

**Authors:** Mengya Yuan, Heqiang Lian, Pan Li

**Affiliations:** 1 Henan Engineering Research Center of Health Big Data and Intelligent Computing, School of Public Health, Institutes of Health Central Plains, Xinxiang Medical University, Xinxiang, Henan, P.R. China; 2 Predictive Toxicology Branch, Division of Translational Toxicology, National Institutes of Environmental Health Sciences, National Institutes of Health, Durham, NC, United States of America; University of Minnesota, UNITED STATES

## Abstract

Sudden cardiac death (SCD) is a leading cause of death worldwide, and the majority of SCDs are caused by acute ventricular arrhythmias (VAs). Early afterdepolarizations (EADs) are an important trigger of VA under pathological conditions, e.g., inherited or acquired long QT syndrome (LQTS). However, it remains unclear how EAD events at the cellular level are spatially organized at the tissue level to induce and maintain ventricular arrhythmias and whether the spatial-temporal patterns of EADs at the tissue level are associated with abnormal T-wave morphologies that are often observed in LQTS, such as broad-based, notched or bifid; late appearance; and pointed T-waves. Here, a tissue model of the Purkinje-ventricular system (PVS) was developed to quantitatively investigate the complex spatial-temporal dynamics of EADs during T-wave abnormalities. We found that (1) while major inhibition of I_CaL_ can substantially reduce the excitability of the PVS leading to conduction failures, moderate I_CaL_ inhibition can promote occurrences of AP alternans at short cycle lengths (CLs), and EAD events preferentially occur with a major reduction of I_Kr_ (>50%) at long CLs; (2) with a minor reduction of I_CaL_, spatially synchronized steady-state EAD events with inverted and biphasic T-waves can be “weakened” into beat-to-beat concurrences of spatially synchronized EADs and T-wave alternans, and as pacing CLs increase, beat-to-beat concurrences of localized EADs with late-appearing and pointed T-wave morphologies can be observed; (3) under certain conditions, localized EAD events in the midmyocardium may trigger slow uni-directional electric propagation with inverted (antegrade) or upright (retrograde) broad-based T-waves; (4) spatially discordant EADs were typically characterized by desynchronized spontaneous onsets of EAD events between two groups of PVS tissues with biphasic T-wave morphologies, and they can evolve into spatially discordant oscillating EAD patterns with sustained or self-terminated alternating EAD and electrocardiogram (ECG) patterns. Our results provide new insights into the spatiotemporal aspects of the onset and development of EADs and suggest possible mechanistic links between the complex spatial dynamics of EADs and T-wave morphologies.

## Introduction

Sudden cardiac death (SCD) is a leading cause of death worldwide, and the majority of SCDs are caused by acute ventricular arrhythmias (VA) [1, 2]. Early afterdepolarization (EAD) is the oscillation and depolarization activity of the membrane potential during action potential (AP) repolarization and represents an important trigger for VA under pathological conditions, such as inherited or acquired long QT syndrome (LQTS) [3]. By decreasing outward currents, e.g., the rapid delayed rectifier potassium current (I_Kr_), AP prolongation with accentuated dispersion of repolarization may give rise to EAD events originating from both Purkinje and ventricular cells in a rate-dependent manner [4], and it has been suggested that the interplay between the branching Purkinje network and ventricular tissue might play an important role in the onset and development of VA [5]. Extensive experimental or computational studies have provided mechanistic insights into the genesis of EAD at both the cellular and tissue levels [6–9]; however, it remains unclear how EAD events at the cellular level are spatially organized at the tissue level to induce and maintain ventricular arrhythmias in the Purkinje ventricular system (PVS).

Transmural dispersion of repolarization (TDR) refers to the difference in the repolarization time between midmyocardial (M) and epicardial (Epi) cells, and such a difference leads to the appearance of T-waves in electrocardiogram (ECG) recordings [10]. It was suggested that the repolarization of Epi cells corresponds to the peak of the T-wave and that of M cells corresponds to the end; therefore, the T_peak-end_ (T_pe_) interval has been proposed as the TDR index [11–14]. Although interval-based indices, e.g., QTc or T_pe_ interval, have been used in clinical practice for the risk stratification of VA, these indices neglect important information regarding spatiotemporal dispersion of repolarization present in abnormal T-wave morphologies [15], e.g., broad-based, notched or bifid, late appearance and pointed T-wave morphologies that are typically observed in LQTS patients with cardiac channelopathies in KCNQ1 (LQT1), KCNH2 (LQT2) or SCN5A (LQT3) [16]. Quantitative analysis of T-wave morphologies to evaluate flatness, notching, asymmetry and restitution properties has been proposed to provide complementary information to the QTc interval in patients with LQTS or heart failure [15, 17]. However, a mechanistic link between spatial-temporal patterns of EADs at the tissue level and abnormal T wave morphologies has not been established. In this study, we developed a tissue model of the PVS to provide mechanistic insights into the onset and development of complex spatiotemporal patterns of EADs and to investigate the relationship between the spatiotemporal dynamics of EAD events and abnormal T wave morphologies.

## Methods

**Fig 1:**
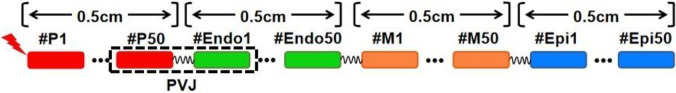
Model schematic of the one-dimensional (ID) Purkinje-ventricular system (PVS) tissue model. Tissue specific conduction velocities and the conduction delay across the Purkinje-ventricular junction (PVJ) were adjusted according to experimental measurements [19, 20].

A one-dimensional (1D) tissue model of the canine PVS (~2 cm in length) was developed by electrically coupling canine Purkinje (P), endocardial (Endo), M and Epi tissue layers (each layer with 50 cells; 200 coupled cells in total) via gap junctions [18] (Fig 1). More specifically, the propagation of electric excitation in the 1D PVS model can be described by the following nonlinear partial differential equation:

$$\frac{\partial V}{\partial t}=\nabla (D\nabla V)-I_{ion}$$

where V (mV) is the membrane potential, D is the diffusion coefficient tensor and I_ion_ is the total ionic current density. To introduce electric and structural heterogeneities into our model, D was calibrated to achieve tissue-specific electric conductions velocities (CV) (Purkinje: CV = 2M/s, D_P_ = 1.2mm^2^/ms; ventricular: CV = 0.5M/s, D_v_ = 0.1mm^2^/ms) as experimentally observed in canine Purkinje and ventricular preparations respectively [19]. At the Purkinje-ventricular junction (PVJ; Fig 1), an electric ratio factor (E_R_) was introduced to phenomenologically account for asymmetrical electric and structural heterogeneities between the quasi-1D Purkinje fiber and the three-dimensional (3D) ventricular myocardium, where D_Pv_ = E_R_*D_v_ with E_R_ = 3.13 to incur a PVJ conduction delay of 4.364ms that is consistent with earlier experimental findings [20]. I_ion_ for different cell types (P, Endo, M and Epi) in our 1D PVS model were derived from previously published canine Purkinje [4] and ventricular [21] cell models based on experimental measurements of transmural electrophysiological heterogeneities in I_NaL_, I_to1_, I_Ks_ and I_NaCa_ [22]. These models were developed to quantitatively represent key characteristics of intracellular Ca^2+^ cycling and membrane ionic currents specific to each cell type using similar modeling and validation approaches [4, 21]. Extracellular unipolar potentials (as pseudo-ECG signals) were computed at a site 2.0 cm away from the epicardium along the strand axis as previously described [18]. Standard programming language C was used. The forward Euler method with an adaptive time step was used for numeric integration. Model codes are available at https://github.com/drpanli/PVS1D.

To induce diverse spatiotemporal patterns of EAD, I_Kr_ and L-type calcium channels (I_CaL_) were randomly inhibited at different levels (ranging from 0%, 10%… 100%), resulting in a total of 121 groups of dual-channel inhibition settings. The random scaling of a pair of major inward (I_CaL_) and outward (I_Kr_) ion channels was used to induce ionic perturbations at different phases of membrane action potential, and maximize the parameter space for complex spatial patterns of EAD to occur. With each group of settings, electric stimuli were applied at the Purkinje end of the tissue to trigger excitation at physiological pacing rates ranging from 1 Hz to 4 Hz (with a 50 ms interval in pacing cycle length (CL); 16 CLs in total), and the last two beats from each simulation were visualized as spatiotemporal maps color-coded according to the membrane potentials; simulated data sets are available at https://github.com/drpanli/PVS1D. The QT interval was defined as the time difference between the electric stimulation and the last cell reaching complete repolarization, and T_pe_ was defined as the time difference between the first and last cell reaching complete repolarization.

## Results

**Fig 2:**
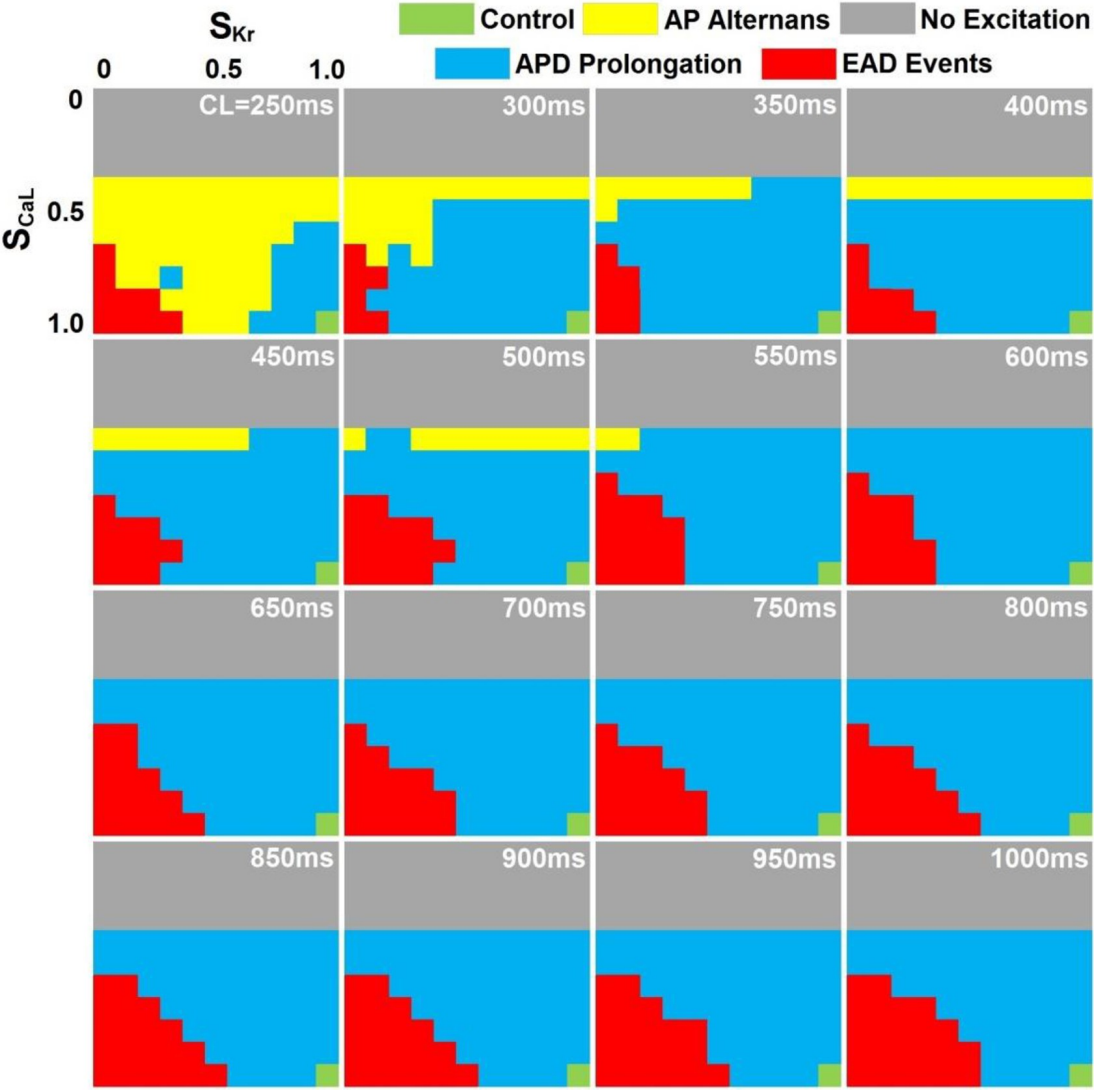
Parameter space mapping of S_Kr_, S_CaL_ and CL, color-coded according to spatiotemporal excitation patterns of the PVS (control: green; gray: no excitation; blue: action potential (AP) prolongation; yellow: AP alternans without early afterdepolarization (EAD) events; red: EAD events). S_Kr_: the scaling factor of I_Kr_ inhibition; S_CaL_: the scaling factor of I_CaL_ inhibition; CL: pacing cycle length.

**Fig 3:**
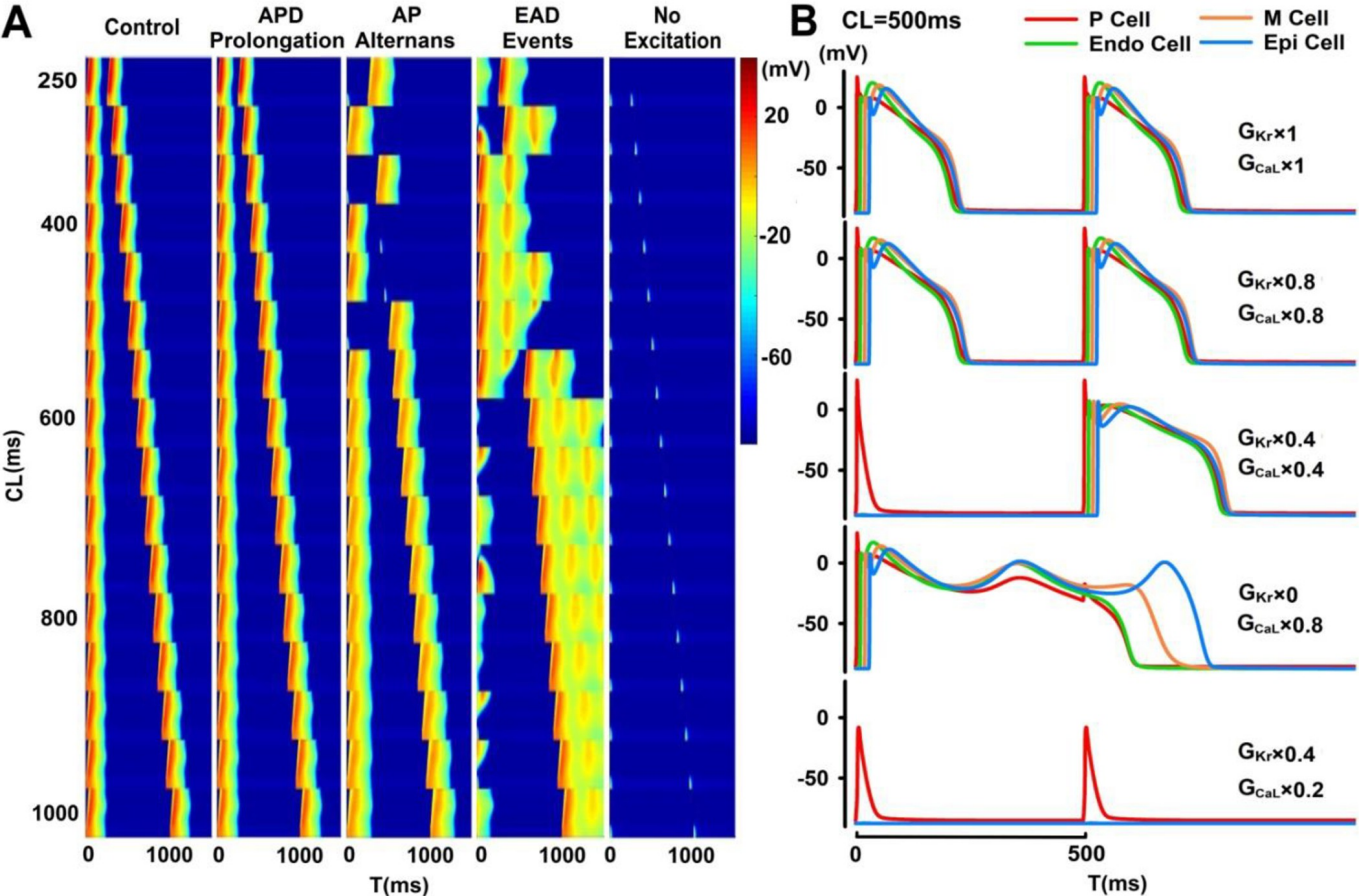
(A) Representative groups of simulations of the PVS tissue model with minor changes, AP alternans, EAD events or no excitation, where the group of simulations were visualized as a stack of 16 spatiotemporal maps with pacing CLs ranging from 250 ms (top) to 1000 ms (bottom). (B) Representative AP morphologies of each cell type at CL = 500 ms under control (top) and pathological conditions.

Our simulation results (121 group of dual-channel inhibition settings, 16 CLs; 1936 simulations in total) can be categorized into control (16 cases), AP prolongation (864 cases), AP alternans (110 cases), EAD events (242 cases) or no excitation (704 cases). In Fig 2, the three-dimensional (3D) parameter space defined by pacing CLs and concurrent blockade of I_CaL_ and I_Kr_ was color-coded according to excitation patterns observed in the PVS. While moderate I_CaL_ blockade (≤60%) can promote occurrences of AP alternans at short CLs, no electric propagation and excitation can be observed with major inhibition of I_CaL_ (≥70%); EAD events preferentially occur with major blockade of I_Kr_ (≥50%) at long CLs. In Fig 3A, representative subtypes of simulations were visualized as stacks of 16 two-dimensional (2D) spatiotemporal maps of membrane potential with pacing CL ranging from 250 ms to 1000 ms. In Fig 3B, selected AP morphologies of P, Endo, M, and Epi cells (located in the center of each tissue layer) at CL = 500 ms were plotted. Under control conditions, electric stimuli trigger robust antegrade excitation under all pacing CLs with AP restitution and morphologies, depolarization and repolarization sequences, consistent with experimental findings [12]. With partial concurrent blockade (20%) of I_Kr_ and I_CaL_, few changes in the excitation patterns can be observed, while major concurrent blockade (60%) of I_Kr_ and I_CaL_ can induce beats to beat “all or nothing” excitation patterns at pacing CL<500 ms. With the complete inhibition of I_Kr_ and partial blockade of I_CaL_ (20%), abundant EAD events with complex spatiotemporal patterns can be observed at all pacing CLs.

### Spatially synchronized EAD events

**Fig 4:**
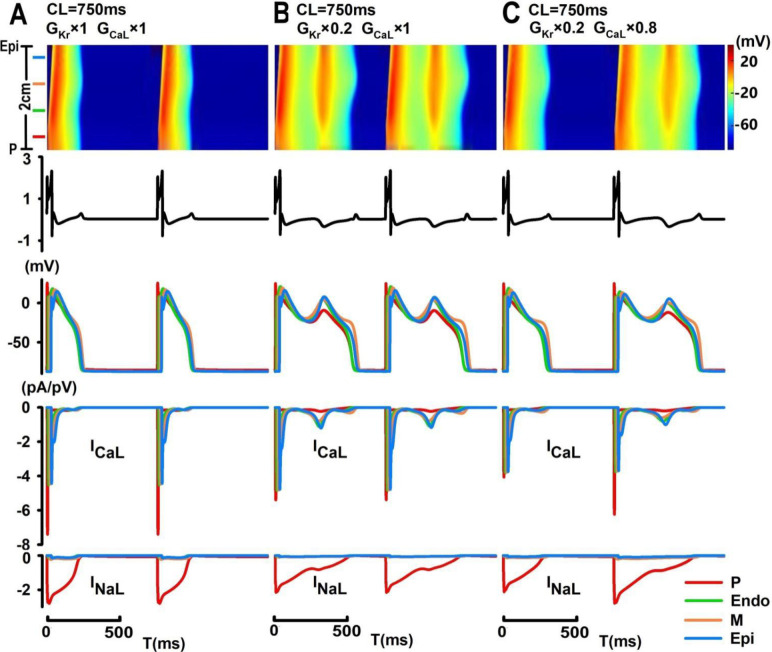
Computer simulation of spatially synchronized EAD events in the PVS tissue model. (A) Normal antegrade excitation of the PVS under control conditions; (B) spatially synchronized steady-state EAD events with T-wave inversion can be induced by an 80% reduction in I_Kr_ at CL = 750 ms and degraded into beat-to-beat occurrences of spatially synchronized EADs and T-wave alternans with an additional blockade of I_CaL_ (20%) (C).

In Fig 4A, under control conditions, electric stimuli can trigger normal antegrade excitations at CL = 750 ms with a QT interval of 247.2 ms and a T_pe_ of 20.4 ms. AP morphologies and underlying ionic currents (I_CaL_ and I_NaL_) of selected cells (indicated as colored lines) were plotted to provide additional insights into spatiotemporal maps of excitation. At the same CLs (750 ms), a major reduction in I_Kr_ (80%) induced substantial AP prolongation and spatially synchronized EAD events with inverted and biphasic T-waves (QT = 566.4 ms; T_pe_ = 44.4 ms) (Fig 4B). During spatially synchronized EAD events, all types of tissues simultaneously developed EAD upstrokes at -22 mV, with different EAD amplitudes (31.18 mV in Epi cells; 15.11 mV in P cells). These synchronized EAD events were identified when EAD upstrokes can be observed in all cell types with minimal time delay comparing to other EAD events observed in our simulation. When combined with a minor reduction in I_CaL_ (20%), the effects of an 80% reduction in I_Kr_ were “weakened” to induce beat-to-beat occurrences of synchronized EADs and T-wave alternans (QT = 323.2 ms, 596.2 ms; T_pe_ = 36.0 ms, 32.8 ms) (Fig 4C).

### Localized EAD events

**Fig 5:**
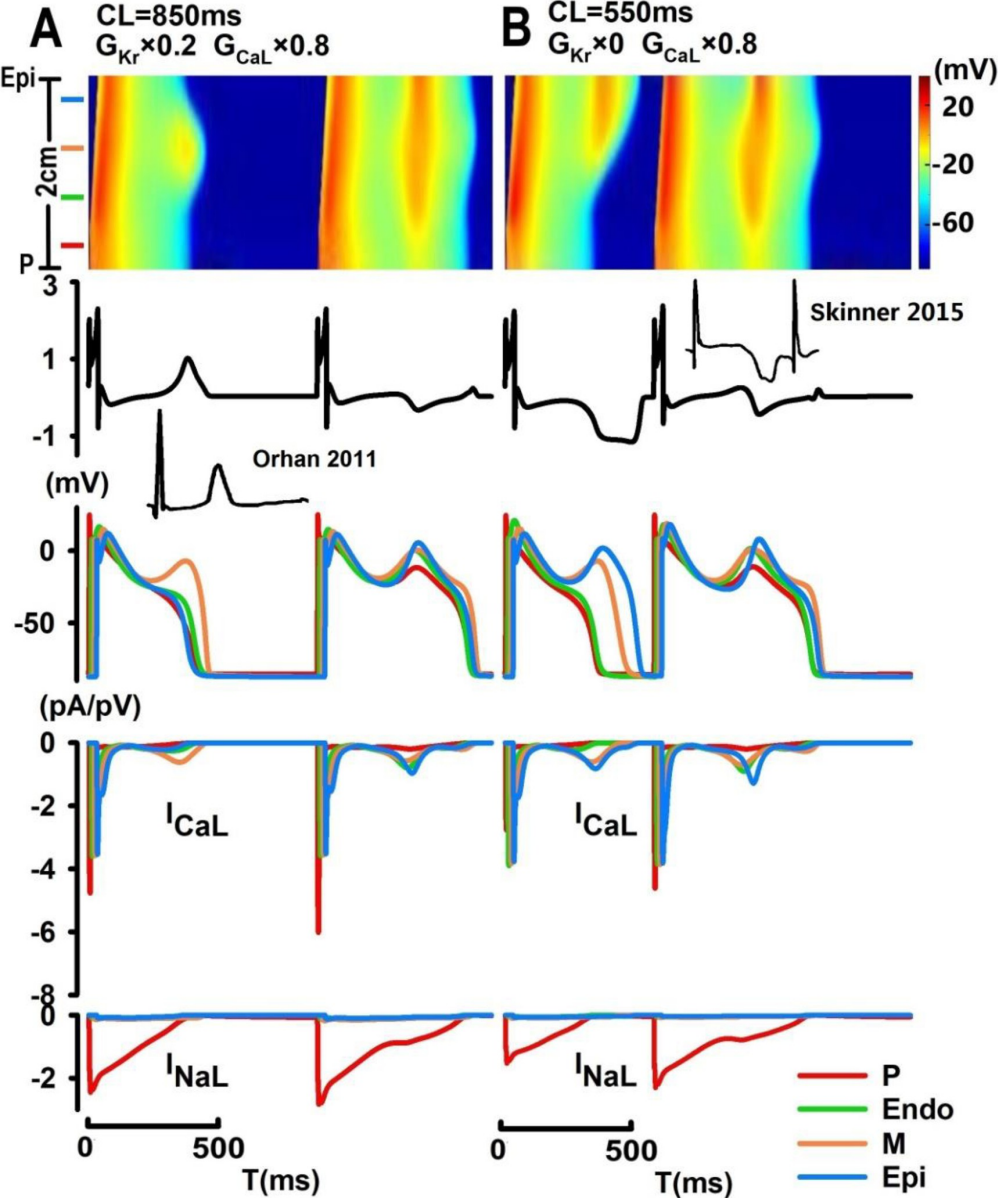
Computer simulation of localized EAD events. (A) With an 80% reduction in I_Kr_ and a 20% reduction in I_CaL_, localized EAD can arise exclusively from the midmyocardium at CL = 850 ms, with a late-appearing and pointed T-wave; (B) with complete inhibition of I_Kr_ and a 20% reduction in I_CaL_, localized EAD can arise simultaneously in the mid- and epicardium, with an inverted broad-based T-wave.

Interestingly, as pacing CLs increase (from 750 ms to 850 ms; Fig 5A), localized EAD events may exclusively arise from midmyocardial tissues to significantly prolong QT (443.2 ms; ↑37%) and T_pe_ (82.4 ms; ↑129%) intervals, with late-appearing (QT prolongation) and pointed (exclusive EADs in the midmyocardium) T-wave morphology [23] and T-wave alternans with reduced beat-to-beat variations in QT intervals (QT = 443.2 ms, 596.9 ms; ↓47%) and increased beat-to-beat differences in T_pe_ (T_pe_ = 82.4 ms, 33.6 ms; ↑1425%). In Fig 5B, under certain conditions (complete inhibition of I_Kr_ and 20% reduction of I_CaL_ at CL = 550 ms), localized EAD events can synchronously arise from two neighboring tissue types, i.e., M and Epi tissues and led to inverted, asymmetric broad-based T-wave morphology [24] in the first beat (QT = 504.8 ms; T_pe_ = 172.8 ms) and T-wave alternans with substantial beat-to-beat variations in T_pe_ intervals (T_pe_ = 172.8 ms, 43.2 ms).

### Localized-EAD-induced unidirectional propagation

**Fig 6:**
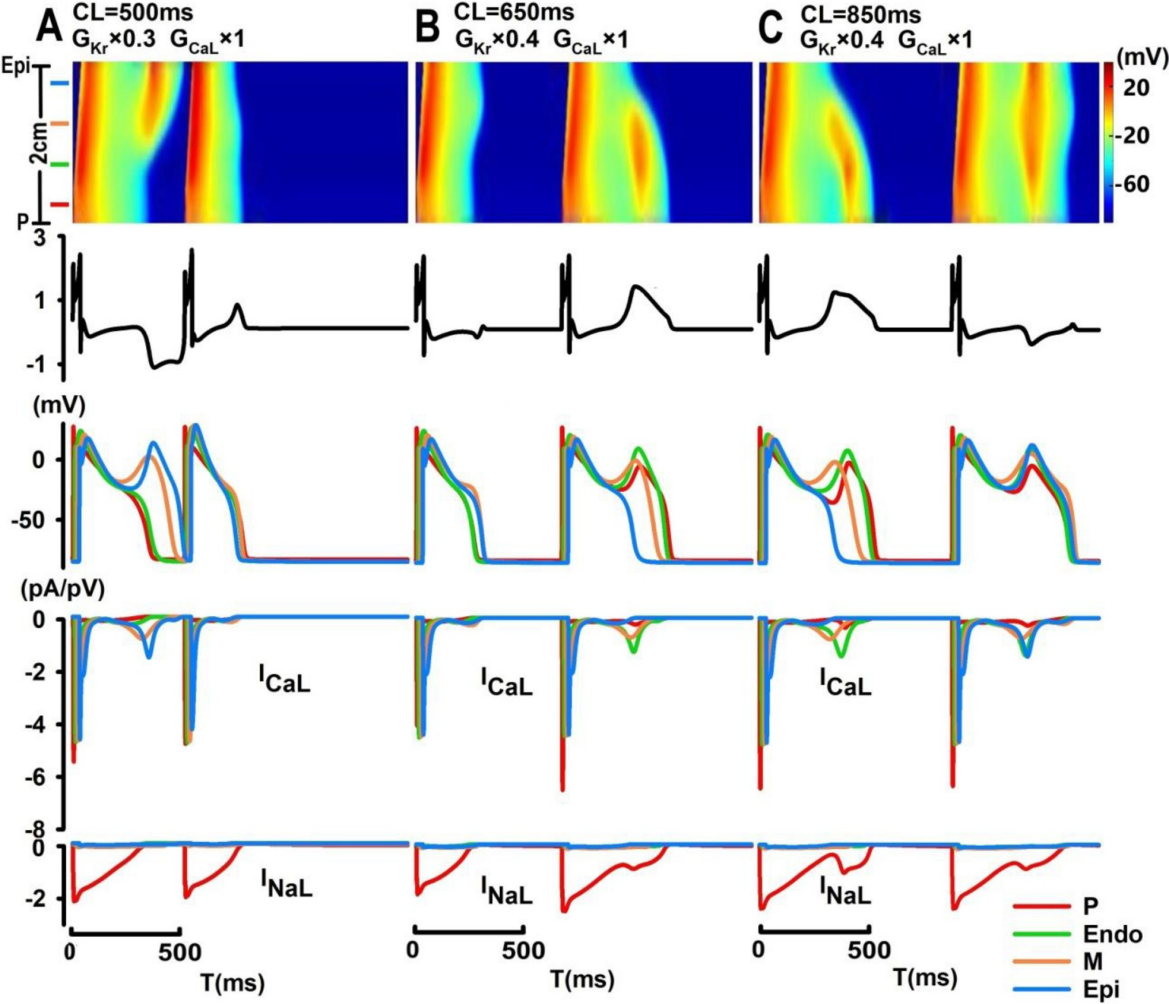
Computer simulation of EAD-induced unidirectional electric propagation. Localized EAD arising from the midmyocardium can trigger spontaneous antegrade propagation with a 70% blockade of I_Kr_ (CL = 500 ms) (A) and spontaneous retrograde propagation with a 60% blockade of I_Kr_ at CL = 650 ms (conduction velocity (CV) = 0.2 m/s; (B)) and at CL = 850 ms (CV = 0.1 m/s; (C)).

In Fig 6, localized EAD events can act as a focal source of arrhythmogenic and trigger spontaneous antegrade or retrograde excitations in the PVS. With a 70% reduction in I_Kr_ at CL = 500 ms, EAD can spontaneously arise from the midmyocardium and trigger unidirectional electric propagation towards the epicardium with a time delay in the onset of EAD and reactivation of I_CaL_ between the M and Epi cells (Fig 6A), leading to an inverted and notched T-wave (QT = 503.6 ms; T_pe_ = 162.9 ms). In Fig 6(B) and 6(C), with 60% reduction of I_Kr_, spontaneous EAD in the midmyocardium (beat #2) can trigger retrograde excitation in the PVS at CL = 650 ms with a conduction velocity (CV) of 0.2 m/s, and sequential development of EAD upstrokes from M cells to Endo and P cells (Fig 6B) with a broad-based asymmetric T-wave (QT = 488.3 ms; T_pe_ = 172.4 ms); at a slower pacing rate (CL = 850 ms), a much slower spontaneous retrograde conduction (CV = 0.1 m/s) can be observed (beat #1) with an increase in both QT interval (514.4 ms) and T_pe_(192.0 ms) and a broad-based T-wave.

### Spatially desynchronized EAD events and oscillatory excitation patterns

**Fig 7:**
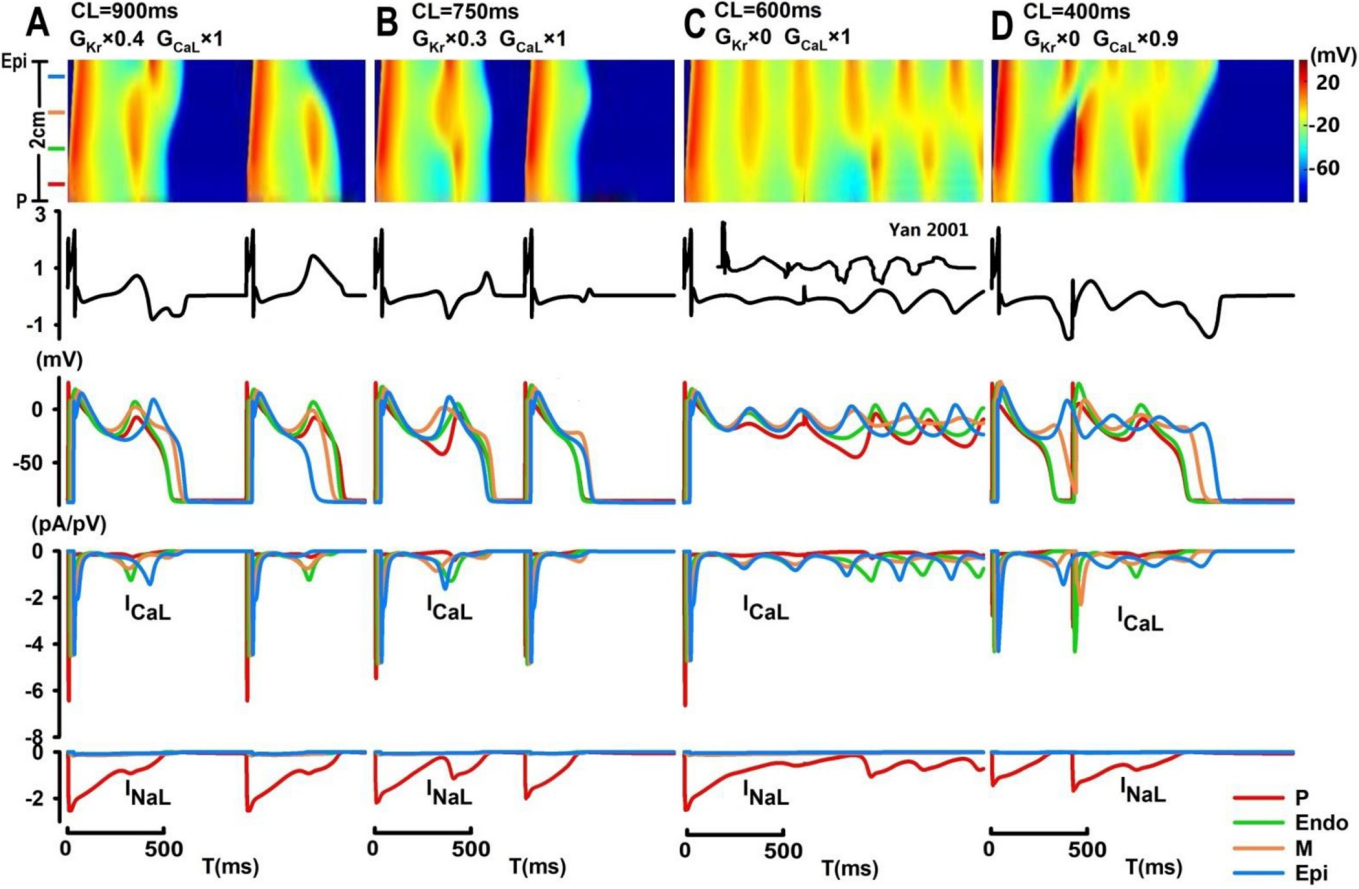
Computer simulation of spatially desynchronized EAD events and oscillatory excitation patterns. (A) Partially synchronized EAD development in P, Endo and M cells preceded the beat-to-beat spontaneous onset of EAD in the epicardium; (B) focal EAD arising from the midmyocardium triggered bidirectional excitation with a late-appearing spontaneous onset of EADs in the P tissue; (C) spatially discordant development of oscillating EAD and ECG patterns with sustained alternating EAD occurrences in the M- and epicardium or in the Purkinje tissue and endocardium; (D) localized EAD events in the epicardium during previous repolarization collided with the current wavefront of depolarization and triggered self-terminated oscillatory EAD and ECG patterns.

In addition to spatially synchronized EAD events, as seen in Fig 4(B) and 4(C), spatially discordant development of EADs can also be induced under certain conditions, typically with a biphasic T-wave morphology. In Fig 7(A), with 60% I_Kr_ inhibition at CL = 900 ms, partially synchronized EAD development in P, Endo and M cells (beat #1) preceded the beat-to-beat spontaneous onset of EAD in the epicardium by 90 ms, with no EAD-induced electric propagation from M to Epi cells, as seen in Fig 6(A). These spatiotemporal excitation patterns give rise to interesting spatial T-wave alternans with alternating biphasic (QT = 594.4 ms; TDR = 83.2 ms; with notching) and monophasic (QT = 489.6 ms; TDR = 168.0 ms; broad-based) T-wave morphologies. With 70% I_Kr_ inhibition at CL = 750 ms (Fig 7B), focal EAD arising from the midmyocardium triggered bidirectional excitations towards both the epi- and endocardium with a late-appearing spontaneous onset of EADs in the P cells, leading to a biphasic T-wave without notching.

In Fig 7(C), complete inhibition of I_Kr_ (CL = 600 ms) can induce severe and complex spatiotemporal patterns of EADs in all tissue types. The first electric stimulus successfully triggered antegrade propagation of excitation with spatially synchronized EADs in the Endo-, M- and epicardium; after the application of the second electric stimulus, spatially discordant development of oscillating EAD waveforms with alternating EAD occurrences in the M- and epicardium or in the Purkinje tissue and endocardium was observed, leading to focal reentrant behaviors and a sustained oscillatory ECG pattern typically observed during ventricular tachycardia [25]. In Fig 7(D), with a complete blockade of I_Kr_ and a 10% reduction in I_CaL_ (CL = 400 ms), localized EAD events in the epicardium during previous repolarization may collide with the current wavefront of depolarization and trigger self-terminated oscillatory EAD patterns. Two electric stimuli (CL = 400 ms) were applied during the simulation, and while two complete APs could be observed in the Purkinje tissue (stimulus-to-response ratio of 2:2), there was only one single AP with oscillatory EADs in the epicardium (stimulus-to-response ratio of 2:1).

## Discussion

In this paper, we presented a 1D tissue model of the PVS and investigated a wide range of complex spatiotemporal patterns of EAD events by exploring the three-dimensional parameter space defined by I_Kr_, I_CaL_ and pacing CLs. Our results suggested that while major inhibition of I_CaL_ (>60%) can substantially reduce the excitability of the PVS and cause conduction failures, moderate I_CaL_ inhibition can promote occurrences of AP alternans at short CLs; EAD events preferentially occur with a major reduction in I_Kr_ (≥50%) at long CLs. With a minor reduction in I_CaL_, spatially synchronized steady-state EAD events with inverted and biphasic T-waves can be “weakened” into beat-to-beat concurrences of spatially synchronized EADs and T-wave alternans. In Fig 4(B), EAD events observed in M, Endo and Epi cells were mostly driven by their intrinsic ion channel dynamics, with secondary electrotonic effects on the onset and amplitude of EADs in these cell types; however, EAD events in P cells were mostly a result of electric coupling to ventricular tissue, given the reactivation of both I_NaL_ and I_CaL_ currents in P cells were much weaker comparing to those shown in Figs 6(B) and 7(C). As pacing CLs increase, beat-to-beat concurrences of localized EADs (e.g., in the midmyocardium) can be observed with late-appearing and pointed T-wave morphologies. Under certain conditions, these localized EAD events in the midmyocardium can act as a focal source of arrhythmogenicity and trigger slow unidirectional electric propagation with inverted (antegrade) or upright (retrograde) broad-based T-waves. In contrast to localized EAD-triggered electric propagation, spatially discordant EADs are typically characterized by desynchronized spontaneous onset of EAD events between different groups of tissues with biphasic T-wave morphologies, and they can evolve into spatially discordant oscillating EAD patterns with sustained or self-terminated alternating EAD and ECG patterns.

Earlier in silico studies using rabbit ventricular cell models, introduced tissue-level heterogeneities by randomly varying the maximum channel conductance or gating variables of a given membrane ion channel [26, 27]. In this study, transmural heterogeneities were modeled based on canine-specific ion channel profiling measurements [22], thus our simulation results could be more clinically relevant given less pronounced electrophysiologic differences between canine and human ventricular myocytes [28]. Recent experimental and computational studies by Liu et al mechanistically linked T-wave alternans to the genesis of ventricular arrhythmias in rabbit models of LQT2 [29]. Instead of interrogating an ion channel mutation associated with a given clinical phenotype, our study highlighted the role of diverse spatial patterns of EAD at tissue level in shaping distinctive T wave morphologies, using a parameter space mapping approach by randomly scaling an inward (I_CaL_) and outward (I_Kr_) ionic current pair. More specifically, our results link localized EAD events, localized EAD-induced unidirectional propagation of excitation and desynchronized EAD events, to late appearing pointed, broad-based, and biphasic T-wave morphologies respectively. These findings provide additional insights into the spatiotemporal aspects of the onset and development of EADs and suggest possible mechanistic links between the complex spatial dynamics of EADs and T-wave morphological patterns.

T-waves, or ECG signals in general, are high-level abstractions of the electric behaviors of a 3D complex biological system (i.e., the mammalian heart), and there can be diverse multiscale mechanisms underlying common T-wave morphologies [30]. It should be noted that our study is limited by the 1D nature of the PVS model, which is an over-simplification of the intrinsic anatomical complexities of the PVS, e.g., the branching Purkinje network, structural heterogeneities at the PVJ, fiber orientations in the myocardium. Our simulation results regarding T-wave morphologies neglected the contribution of apicobasal and left-right heterogeneities of the heart [31]. Further development of human-specific systems models of the PVS [32] is required to simulate complex interactions between the human Purkinje network and ventricular systems and to provide computational tools to accelerate the prevention and treatment of lethal ventricular arrhythmias.
